# Influenza season influence on outcome of new nodules in the NELSON study

**DOI:** 10.1038/s41598-023-33672-4

**Published:** 2023-04-21

**Authors:** H. L. Lancaster, M. A. Heuvelmans, G. H. de Bock, Y. Du, F. A. A. Mohamed Hoesein, K. Nackaerts, J. E. Walter, R. Vliegenthart, M. Oudkerk

**Affiliations:** 1 Department of Epidemiology, University of Groningen, University Medical Center Groningen, Groningen, The Netherlands; 2 Department of Radiology, University Medical Center Utrecht, Utrecht University, Utrecht, The Netherlands; 3 Department of Pneumology, University Hospital Leuven, KU Leuven, Leuven, Belgium; 4 Department of Medical Oncology and Hematology, University Hospital Zurich, Zurich, Switzerland; 5 Department of Radiology, University of Groningen, University Medical Center Groningen, Groningen, The Netherlands; 6 Faculty of Medical Sciences, University of Groningen, Antonius Deusinglaan 1, 9713 AV Groningen, The Netherlands

**Keywords:** Cancer screening, Lung cancer

## Abstract

We evaluated the impact of the influenza season on outcome of new lung nodules in a LDCT lung cancer screening trial population. NELSON-trial participants with ≥ 1 new nodule detected in screening rounds two and three were included. Outcome (resolution or persistence) of new nodules detected per season was calculated and compared. Winter (influenza season) was defined as 1st October to 31st March, and compared to the summer (hay-fever season), 1st April to 30th September. Overall, 820 new nodules were reported in 529 participants. Of the total new nodules, 482 (59%) were reported during winter. When considering the outcome of all new nodules, there was no statistically significant association between summer and resolving nodules (OR 1.07 [CI 1.00–1.15], *p* = 0.066), also when looking at the largest nodule per participant (OR 1.37 [CI 0.95–1.98], *p* = 0.094). Similarly, there was no statistically significant association between season and screen detected cancers (OR 0.47 [CI 0.18–1.23], *p* = 0.123). To conclude, in this lung cancer screening population, there was no statistically significant association between influenza season and outcome of new lung nodules. Hence, we recommend new nodule management strategy is not influenced by the season in which the nodule is detected.

## Introduction

Lung cancer remains the most common cause of cancer related deaths in the world. In 2020, it accounted for 1.8 million deaths worldwide^1–3^. Therefore, in the United States it is highly recommended high-risk individuals undergo low-dose CT screening for lung cancer^4–11^. However, many European countries are still debating whether to recommend low-dose CT screening to high-risk individuals, despite the recent publication of the positive results from the largest European lung cancer screening trial^12,13^. Only a small number of countries, such as the United Kingdom and Poland, have introduced lung cancer screening programs. In 2003, the aforementioned Dutch-Belgium Randomised Lung Cancer Screening trial (NELSON) was launched^14^. The final results have shown that low-dose CT lung cancer screening can reduce lung cancer mortality by 24% in males and 33% in females, in high risk ex-smokers aged 50 to 75 years old^13,15^.

A drawback in lung cancer screening is the high rate of false-positive screen results caused by the large number of (mostly benign) lung nodules detected. This high false-positive rate has already been reduced through the use of volumetric measurement and volume doubling time (VDT) follow-up, however for the optimisation of lung cancer screening, false-positive results should be as low as feasibly possible^16^. A sub-study of the NELSON trial has shown solid new nodules have a significantly higher risk of malignancy compared to baseline nodules, and are detected at each screening in around 5–7% of subjects^17^. Therefore, more attention is now being paid to the new nodules detected^17,18^. We know that a large part of the benign new nodules disappear at follow-up CT imaging, however factors predicting resolution have –until now– not been identified.

In a previous study of the general Dutch population, a seasonal variation was found in the presence and characteristics of LDCT-detected lung nodules^19^. Significantly more baseline lung nodules were detected in the summer months (April to September) when hay fever is most prevalent (56% in summer versus 44% in winter; OR 1.255, *p* = 0.002), and a seasonal variation was seen in lung nodule characteristics. More spherical nodules were detected in summer whereas more polygonal and irregular nodules were detected in winter. In the winter, more nodules were located in the left upper lobe, and there were significantly more atypical perifissural nodules detected^19^. This study however only included baseline nodules and no nodule follow-up or pathological outcome data was available, and it was not a lung cancer screening trial.

Research has shown there are a greater number of hospitalisations during winter for respiratory diseases than any other season^20^. In the Netherlands and Belgium there is an influenza outbreak in winter almost every year for approximately 14 weeks. Cases have been reported from October onwards, with the peak incidence being between December and March^21,22^. Since it is expected that most of the resolving new nodules are infectious, it can be hypothesized that more new nodules will be found during the winter influenza season, with a higher rate of these nodules resolving. The Netherlands and Belgium have an almost identical climate across all provinces due to the relatively small size of the countries. Therefore, this study is unique in that the climate for each season is the same for all provinces.

Our study therefore aims to evaluate the impact of the season of the year when respiratory illnesses are most prevalent, winter (influenza season), on outcome of new lung nodules in NELSON-trial participants.

## Methods

### Study and participants

The NELSON trial study design and participant recruitment has been documented numerous times before^14,17,18^. In short, the NELSON trial began in December 2003 and 15 822 participants from the Netherlands and Belgium were registered up until July 2006. The participants were each assigned to receive low-dose screening (n = 7915) or no screening (n = 7907) at random. The eligibility and exclusion criteria have been published previously^14^.

Baseline screening (year one) was carried out between April 2004 and December 2006. Follow-up screening was carried out in years two (second round), four (third round) and six (fourth round), after a 1-year, 2-year and 2.5-year interval respectively. For this study, we identified all participants who developed a new resolving or persisting solid or subsolid lung nodule during screening rounds two and three, registered by NELSON radiologists as new or smaller than 15mm^3^ (study detection limit) at previous screening rounds. For this, we used the NELSON management system in which information of all NELSON scans was stored. The NELSON trial was approved by Ethics Committees of all participating centres in the Netherlands and Belgium (University Medical Centre Groningen, University Medical Centre Utrecht, Kennemer Gasthuis Haarlem, and University Hospital Leuven) and authorised by the Dutch Health Care Committee. All participants gave written informed consent. The study was performed in accordance with relevant guidelines/regulations and with the Declaration of Helsinki^17^.

### NELSON screening CT scan protocol, analysis and data management

The CT scan protocol of the NELSON trial has been previously published^14,17,18^. All screening sites had a 16-multidetector or 64–multidetector (later screening rounds) (3 Sensation-16, Siemens Medical Solutions, Forchheim, Germany; and 1 Mx8000 IDT or Brilliance 16P, Philips Medical Systems, Best, Netherlands) and CT scanner and conditions were standardised. Reconstructions were made with 1.0 mm slice width and 0.7 mm interval. CT scans were read by two or more independent radiologists with one to 20 years’ experience in the first two screening rounds, and following rounds were read by a single radiologist with a minimum of six years’ experience. CT scan analysis for semi-automated volume measurement was performed on Siemens Leonardo workstations using the Syngo Lungcare software package (version Somaris/5 VA70C-W, Siemens Medical Solutions)^14^. For subsequent CT scans, nodules were individually matched on previous scans depending on consistency, size, and location (the software’s matching algorithm). If the nodule was not present previously, or was smaller than the detection limit (< 15mm^3^) at previous scan, it was classified as a new nodule^17^.

**Figure 1:**
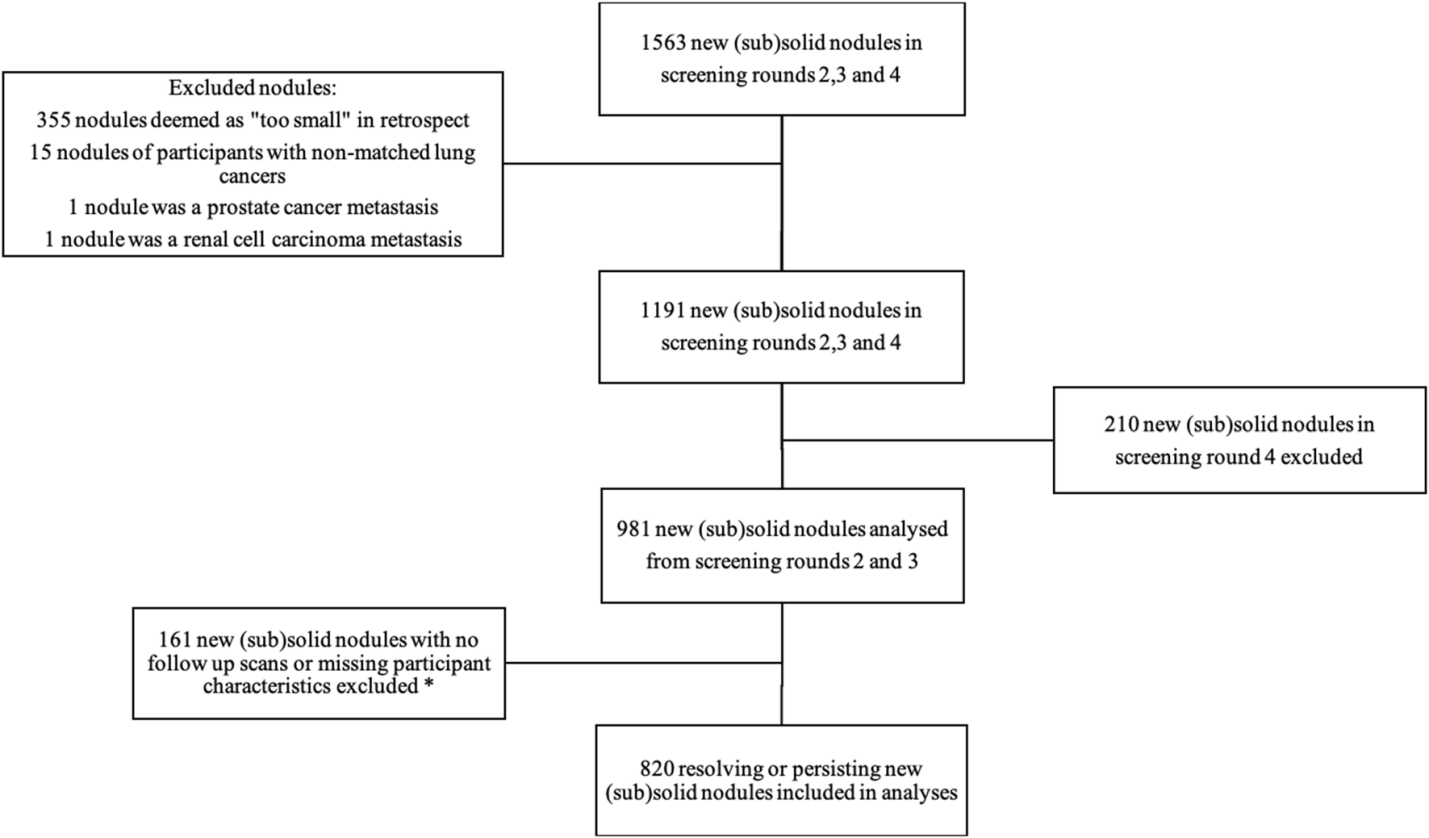
Overview of new resolving and persisting (sub)solid lung nodules included in this sub-study of the NELSON trial. *a nodule had no follow up scan when a participant was referred immediately to a pulmonologist for further diagnostics, or the participant did not require an additional CT scan according to protocol.

This study used information at first nodule detection as reported in the NELSON management system. In this study, we excluded all nodules from screening round four, as this round was a sub-group of the participants who were predominantly current smokers. Additionally, we excluded lung nodules which were either: deemed “too small” (< 15mm^3^) in retrospect, from participants with a non-matched lung cancer or a metastasis (renal and prostrate), and those nodules leading to an immediate referral or no additional screen, or missing participant characteristics. A total of 820 new (sub) solid nodules were analysed, an overview can be seen in Fig. 1.

### Nodule management

The NELSON nodule management protocol has been explained in detail previously^14,17,18^. In short, all solid nodules detected were categorised in one of four ways (NODCAT I, II, III, IV) based on their size and characteristics. NODCAT I was considered negative, NODCAT II and III were considered intermediate, and NODCAT IV was considered positive. Following initial detection of a nodule, subsequent evaluations were based on both the volume doubling time and the growth of the nodule.

### New nodule outcome

For a nodule to be classified as resolving, it should not have been registered on the NELSON management system on the follow-up LDCT. This could be because the radiologist considered the nodule to have disappeared, to be non-measurable, or they registered the nodule as calcified. Resolution of a nodule was considered spontaneous during this trial as no intervening therapy was issued. Further, lung nodules were matched retrospectively to pathologically confirmed screen detected lung cancers.

### Seasonal influence

For this study, we had a specific interest in the winter (influenza season) in comparison to the summer (hay fever season). The Netherlands National Institute for Public Health and the Environment (RIVM) suggests that the yearly influenza epidemic is usually between December and March, with cases recorded as early as October^22^. There is a yearly peak in pollen levels, and subsequently an increased prevalence of hay fever cases, between April and September^23^. Therefore, we decided to compare two seasons; winter (influenza season) defined as 1st October to 31st March, and summer (hay fever) defined as 1st April to 30th September.

### Participant and nodule level analysis

The total number new nodules and number of participants with ≥ 1 new nodule was calculated for rounds two and three combined. To analyse the data on nodule level we looked at the total new nodules detected per month and per season. To analyse the data on participant level, we looked at the largest nodule per participant and excluded any multiple nodules in the same participant. The number of resolving and persisting nodules, and nodules matched to screen detected cancers was calculated.

### Statistical analysis

Descriptive statistics were used to report absolute frequencies and percentages. Binary logistic regression analyses were performed to investigate a possible seasonal influence on resolution of new lung nodules, and screen detected cancers at participant level. Multilevel logistic regression analyses were performed to investigate a possible seasonal influence on resolution of new lung nodules at nodule level. Variables included in the aforementioned analyses included; season (summer and winter), age, gender (male and female), smoking status (former and current). Separate analyses were performed which also included the following nodule characteristics; nodule volume and nodule location (upper and lower lobe), see supplement. In case of missing nodule size or location, those nodules were excluded in the regression analyses.

## Results

### Participant characteristics

There were 1563 new (sub)solid nodules detected in screening rounds two, three and four, as previously reported^24,25^. In our dataset there were 820 new resolving or persisting nodules detected in 529 participants who underwent a LDCT thorax scan in screening rounds two and three of the NESLON trial. An overview of participant characteristics, including distribution based on nodule outcome (resolved versus persisted), can be seen in Table 1. Median participant age was 59 years (inter-quartile-range [IQR] 55–63), 411 (78%) were male, 330 (62%) were current smokers, and the median pack-years was 39 (IQR 30–50). 316 (60%) of individuals (with ≥ 1 resolving or persisting new nodule) were scanned in the winter, and 213 (40%) in the summer.

**Table 1:** Characteristics of participants with ≥ 1 resolving or persisting new nodule reported during screening rounds two and three of the NELSON trial, including distribution based on nodule outcome (resolving versus persisting).

Overall (n = 529)		Nodule outcome (largest nodule)	
		Resolved (n = 327 [62%])	Persisted (n = 202 [38%])
Gender			
Female	118	85 [72%]	33 [28%]
Male	411	242 [59%]	169 [41%]
Age (years)			
≤ 54	110	75 [68%]	35 [32%]
55–64	314	193 [61%]	121 [39%]
≥ 65	105	59 [56%]	46 [44%]
Median (IQR)	59 (55–63)	59 (55–63)	59 (56–64)
Smoking status			
Current	330	209 [63%]	121 [37%]
Former	199	118 [59%]	81 [41%]
Smoking pack-years			
≤ 59	454	280 [62%]	174 [38%]
≥ 60	75	47 [63%]	28 [37%]
Median (IQR)	39 (30–50)	39 (30–50)	39 (30–53)
Season			
Summer	213	139 [65%]	74 [35%]
Winter	316	188 [59%]	128 [41%]

*N* total number of participants with ≥ 1 new lung nodule, *IQR* Inter quartile range. Summer (Hay fever season) was defined as 1st April to 30th September, and winter (Influenza season) as 1st October to 31st March.

### New lung nodule outcomes

Monthly and seasonal distribution of total new nodules, participants with > 1 new nodule and new nodule outcome for both resolving and persisting nodules detected during screening rounds two and three can be found in Table 2. Of the total 820 new resolving and persisting lung nodules, 482 (59%) were reported in winter. When looking at new nodule outcome, 226 (67%) of the new nodules reported in the summer (n = 338) resolved. In winter, 304 (63%) of the new nodules reported were resolving. Similar percentages were seen when looking at the largest nodule per participant. In summer, 139 (65%) resolving nodules were reported in the 213 participants with ≥ 1 new nodule, compared to 188 (60%) resolving nodules reported in 316 participants in winter. In a further nuanced analysis of new solid (780 [95%]) verses new subsolid (40 [5%]) nodules, 457 (59%) of the new solid nodules and 25 (63%) of the new subsolid nodules were detected in winter. The percentage of new resolving solid nodules was equal to the percentage of new resolving subsolid nodules (504 [65%] solid versus 26 [65%] subsolid), and 289 (57%) new resolving solid nodules versus 15 (58%) new resolving subsolid nodules were detected in the winter. Again, similar results were seen when looking at the largest nodule per participant. Number of screen detected cancers on the other hand followed a different trend. A total of 23 screen detected cancers were present in this study population. Of the 213 participants with ≥ 1 new nodule in the summer, 6 (2.8%) had a nodule that was retrospectively matched to a screen detected lung cancer. This was comparatively less than in the winter; 17 (5.4%) of the 316 participants.

**Table 2:** Monthly and seasonal distribution of total new nodules, participants with ≥ 1 new nodule and new nodule outcome for both resolving and persisting nodules, and screen detected lung cancers reported during screening rounds two and three of the NELSON trial.

			Nodule Level (n = 820)		Participant Level (n = 529)		
	New resolving and persisting nodules	Participants with ≥ 1 nodule	Resolved	Persisted	Resolved	Persisted	Screen detected lung cancers
January	73	46	55	18	31	15	0
February	73	50	42	31	27	23	3
March	72	44	41	31	27	17	2
April	52	35	36	16	20	15	1
May	48	31	34	14	22	9	0
June	49	33	36	13	22	11	1
July	55	39	40	15	29	10	0
August	45	22	26	19	15	7	0
September	89	53	54	35	31	22	4
October	91	60	58	33	36	24	2
November	90	63	53	37	33	30	7
December	83	53	55	28	34	19	3
Summer	338 [41%]	213 [40%]	226 [43%]	112 [39%]	139 [43%]	74 [37%]	6 [26%]
Winter	482 [59%]	316 [60%]	304 [57%]	178 [61%]	188 [57%]	128 [63%]	17 [74%]
Total	820	529	530	290	327	202	23

Summer (Hay fever season) was defined as 1st April to 30th September, and winter (Influenza season) as 1st October to 31st March.

When performing logistic regression analyses, summer season does not significantly predict new nodule resolution. This remained true at both nodule and participant level analysis (OR 1.07 [CI 1.00–1.15], *p* = 0.066 and OR 1.37 [CI 0.95–1.98], *p* = 0.094, respectively). Additionally, we observed no significant association between season of year and screen detected cancers (OR 0.47 [CI 0.18–1.23], *p* = 0.123). A more detailed outline of the results can be seen in Table 3.

**Table 3:** Logistic regression analyses of nodule outcome (resolved versus persisted) and screen detected lung cancers during screening rounds two and three of the NELSON trial.

Predictor variables	p-value	OR	95% CI for OR	
			Lower	Upper
Nodule level multilevel logistic regression analysis; Dependant variable: resolved [1]/persisted [0]				
Season (summer)	0.066	1.07	1.00	1.15
Age	0.087	0.99	0.99	1.00
Smoking status (former)	0.593	0.98	0.91	1.06
Gender (female)	0.001	1.16	1.06	1.26
Participant level binary logistic regression analysis; Dependant variable: resolved [1]/persisted [0]				
Season (summer)	0.094	1.369	0.947	1.977
Age	0.256	0.981	0.949	1.014
Smoking status (former)	0.486	0.877	0.607	1.268
Gender (female)	0.008	1.853	1.175	2.924
Participant level binary logistic regression analysis; Dependant variable: screen detected lung cancer yes [1]/no [0]				
Season (summer)	0.123	0.471	0.181	1.225
Age	0.307	1.040	0.964	1.123
Smoking status (former)	0.721	1.171	0.494	2.775
Gender (female)	0.113	0.303	0.069	1.325

(Reference category); OR odds ratio; 95% CI 95% confidence interval for odds ratio.

One variable which was significantly associated with the resolution of new nodules was gender. Female gender was associated with resolution of a new lung nodule at both nodule level analysis (OR 1.16 [CI 1.06–1.26], *p* = 0.001), and participant level analysis (OR 1.85 [CI 1.18–2.92], *p* = 0.008). When nodule location was added to the multilevel regression analysis, a nodule located in the upper lobe was negatively associated with its resolution (OR 0.90 [CI 0.84–0.96], *p* = 0.002). This remained true for the participant level binary regression analysis (OR 0.57 [CI 0.39–0.85], *p* = 0.005), when looking at the largest nodule per participant. Nodule location and screen-detected lung cancer outcome followed a different trend. Albeit non-significant, we observed nodules located in the upper lobe were positively associated with screen detected lung cancer (OR 2.41 [CI 0.86–6.73], *p* = 0.095), see supplementary results for an overview of regression analyses including nodule characteristics.

## Discussion

In this NELSON lung cancer screening trial sub-study, we aimed to investigate whether the season of year when most respiratory illnesses are prevalent, winter (influenza season), had an impact on outcome of new lung nodules detected. We can report, no statistically significant association was found when looking at all new nodules and the largest new nodule per participant in a lung cancer screening population (OR 1.07 [CI 1.00–1.15], *p* = 0.066 and OR 1.37 [CI 0.95–1.98], *p* = 0.094 respectively). Additionally, we saw no statistically significant association between screen-detected lung cancer and the season of year (OR 0.47 [CI 0.18–1.23], *p* = 0.123).

Our findings are not in agreement with what we had hypothesized; that more of the new lung nodules detected during the winter (influenza season) would be resolving due to the increased incidence of respiratory illnesses. Our results would therefore suggest that new nodules detected during the winter (influenza season) require the same careful attention as those detected in summer, as it cannot be concluded that they are simply infectious nodules. Furthermore, as we see a considerable number of resolving nodules during the summer (April to September), we can speculate that hay fever (allergic rhinitis) suffers could present with more new resolving nodules during the hay fever season when taking part in a lung cancer screening trial. Existing research on associations between allergic rhinitis, smoking and lung cancer is limited and often conflicting^26–28^. There have however, been previous links suggesting an association between chronic rhinosinusitis and lung cancer^29,30^. As the upper and lower airway have a similar pathophysiology, it could be speculated that an inflammatory response in the upper airway could trigger an up regulation of the immune response in the lower airway, resulting in the development of lung nodules^31^. As far as we know, no research has looked at a possible connection between participants with allergic rhinitis and the development of new lung nodules and therefore this could be a potential for future research. Additionally, it might be interesting to investigate if more women with allergic rhinitis develop new lung nodules that go on to resolve. We reported female gender was positively associated with the resolution of a new lung nodule when looking at the largest nodule per participant (OR 1.85 [CI 1.18–2.92], *p* = 0.008), however no existing research could be found to explain this outcome.

These findings also support, in part, those reported in the ImaLife study representing the general Dutch population, where presence of lung nodules was increased during the hay fever season on baseline LDCT thorax scans^19^.

The trend we observed between new lung nodule location and screen detected cancers (11 [48%] of the screen detected lung cancers were located in the right upper lung) is supported by existing literature. Horeweg et al. reported 45% of all lung cancers detected in the NELSON trial were located in the right upper lobe^32^. A similar result was seen when a univariate analysis was performed for solid new lung nodules in the NELSON trial: new lung nodules located in the right upper lung were significantly more likely to have lung cancer as an outcome than those in a different location (OR 1.9 [95%CI 1.2–3.1], p = 0.011)^33^. This is a result of unequal distribution of airflow in favour of the right upper lobe during the beginning of inspiration, and henceforth an increased accumulation of tobacco smoke particles^34^. What remains unexplained is the negative association between resolving new nodules and the upper lung lobe. This should be investigated further in future research.

Unsurprisingly, the majority of participants in this study were male and current smokers. This can be explained by the inclusion criteria used in the NELSON trial. Mean age (59 years) however cannot be explained in the same way. Participants in the NELSON trial were aged between 50 and 75 years, hence a higher mean age would also be expected in this study. It can be hypothesized that the younger age of participants with new nodules, the majority of which resolving, is due to an increased immune response to antigens. Research has shown that increasing age is accompanied by immune system remodelling. Changes usually begin in the sixth decade and progress over time to a state of immunosenescence. Therefore, younger participants may be more likely to have a stronger response to antigens leading to the development of inflammatory lung nodules^35,36^.

This study was unique because of the small size of the countries where the study took place. The Netherlands and Belgium have a similar climate throughout the provinces. A study by Hatch found that the mean minimum and maximum monthly temperatures differed by < 0.5 °C nationally^37^. For this reason the climate will be the same for all participants for each month and therefore each season. Additionally, this study added to previously reported seasonal variation research, by also reporting on nodule outcomes. However, a possible limitation to this study is that the exact timing that new nodules appeared is unknown. The date of detection of new nodules may not be the exact date at which the nodule developed. Therefore, a new nodule detected in April; the start of the hay fever season, could have already been present in one of the winter months (influenza season) and vice versa. Furthermore, the sample size of our sub-study could be the reason for the lack of significant findings. By increasing the sample size, and in turn the power of the study, we may have seen a statistically significant association between season and new nodule outcome.

Future research could investigate whether there is a seasonal influence on the outcome of new lung nodules in the general population, but also more specifically in persons who are diagnosed with allergic rhinitis or other inflammatory respiratory conditions.

To conclude, we report that no statistically significant association was found between the winter (influenza) season and the outcome of new lung nodules in this lung cancer screening population. Therefore, underlying pathophysiology of resolving nodules remains uncertain. Hence, we recommend that all new nodules should be treated with the same careful attention irrespective of the season in which they are detected.

## Supplementary Information

Supplementary Information.

## Data Availability

The data used in this sub-study are not publicly available, but are available from the corresponding author on reasonable request.
