# Supplementary material for: Influenza season influence on outcome of new nodules in the NELSON study
Source: Sci Rep. 2023 Apr 21;13:6589. doi: 10.1038/s41598-023-33672-4 (PMC10121576; doi:10.1038/s41598-023-33672-4)
Supplement: Supplementary file 1 — Supplementary Information. [file 41598_2023_33672_MOESM1_ESM.docx]

**Influenza season influence on outcome of new nodules in the NELSON study**

H. L. Lancaster^1^, M. A. Heuvelmans^1^, G.H. de Bock^1^, Y. Du^1^, F. A. A. Mohamed Hoesein^2^, K. Nackaerts^3^, J.E. Walter^4^, R. Vliegenthart^5^, M. Oudkerk^6^.

Affiliations

1. *Department of Epidemiology, University of Groningen, University Medical Center Groningen, Groningen, The Netherlands*
2. *University Medical Center Utrecht, Utrecht University, Department of Radiology, Utrecht, The Netherlands*
3. *Department of Pneumology, University Hospital Leuven, KU Leuven, Leuven, Belgium*
4. *Department of Medical Oncology and Hematology, University Hospital Zurich, Zurich, Switzerland*
5. *Department of Radiology, University of Groningen, University Medical Center Groningen, Groningen, The Netherlands*
6. *Faculty of Medical Sciences, University of Groningen, Groningen, The Netherlands*

*Corresponding author

Prof. Dr. Matthijs Oudkerk

*Faculty of Medical Sciences, University of Groningen, Antonius Deusinglaan 1, 9713 AV Groningen, The Netherlands*

*m.oudkerk@rug.nl*

**Supplementary results**

**Logistic regression analyses of nodule outcome (resolved verses persisted) and screen detected lung cancers during screening rounds two and 3 of the NELSON trial.**

***Nodule level multilevel logistic regression analysis***

Dependant variable: resolved [1]/persisted [0]

| Predictor variables | p-value | OR | 95% CI for OR | |
| --- | --- | --- | --- | --- |
|  |  |  | Lower | Upper |
| Season (summer) | 0.108 | 1.06 | 0.99 | 1.15 |
| Age | 0.071 | 0.99 | 0.99 | 1.00 |
| Smoking status (former) | 0.430 | 0.97 | 0.89 | 1.05 |
| Gender (female) | 0.001 | 1.16 | 1.06 | 1.27 |
| Nodule volume | 0.003 | 1.00 | 1.00 | 1.00 |
| Nodule location (upper lobe) | 0.002 | 0.90 | 0.84 | 0.96 |
| Nodule location (trachea, bronchi, biurfication) | 0.542 | 1.33 | 0.53 | 3.35 |

(reference category); OR *odds ratio*; 95% CI *95% confidence interval for odds ratio*

***Participant level binary regression analysis***

Dependant variable: resolved [1]/ persisted [0]

| Predictor variables | p-value | OR | 95% CI for OR | |
| --- | --- | --- | --- | --- |
|  |  |  | Lower | Upper |
| Season (summer) | 0.122 | 1.366 | 0.920 | 2.026 |
| Age | 0.235 | 0.979 | 0.946 | 1.014 |
| Smoking status (former) | 0.380 | 0.837 | 0.563 | 1.245 |
| Gender (female) | 0.006 | 1.998 | 1.221 | 3.270 |
| Nodule volume | 0.031 | 0.999 | 0.998 | 1.000 |
| Nodule location (upper lobe) | 0.005 | 0.572 | 0.386 | 0.847 |

(reference category); OR *odds ratio*; 95% CI *95% confidence interval for odds ratio*

Dependant variable: screen detected lung cancer yes [1]/no [0]

| Predictor variables | p-value | OR | 95% CI for OR | |
| --- | --- | --- | --- | --- |
|  |  |  | Lower | Upper |
| Season (summer) | 0.083 | 0.400 | 0.142 | 1.126 |
| Age | 0.319 | 1.041 | 0.962 | 1.125 |
| Smoking status (former) | 0.876 | 0.928 | 0.362 | 2.378 |
| Gender (female) | 0.121 | 0.308 | 0.069 | 1.367 |
| Nodule volume | 0.960 | 1.000 | 0.998 | 1.002 |
| Nodule location (upper lobe) | 0.095 | 2.406 | 0.860 | 6.733 |

(reference category); OR *odds ratio*; 95% CI *95% confidence interval for odds ratio*
